# *Shank3* Transgenic and Prenatal Zinc-Deficient Autism Mouse Models Show Convergent and Individual Alterations of Brain Structures in MRI

**DOI:** 10.3389/fncir.2019.00006

**Published:** 2019-02-22

**Authors:** Michael Schoen, Harun Asoglu, Helen F. Bauer, Hans-Peter Müller, Alireza Abaei, Ann Katrin Sauer, Rong Zhang, Tian-jia Song, Juergen Bockmann, Jan Kassubek, Volker Rasche, Andreas M. Grabrucker, Tobias M. Boeckers

**Affiliations:** ^1^Institute for Anatomy and Cell Biology, Ulm University, Ulm, Germany; ^2^Neurology Department, Ulm University, Ulm, Germany; ^3^Core Facility Small Animal MRI, Ulm University, Ulm, Germany; ^4^Department of Biological Sciences, University of Limerick, Limerick, Ireland; ^5^Neuroscience Research Institute, Peking University, Beijing, China; ^6^Department of Neurobiology, School of Basic Medical Sciences, Peking University, Beijing, China; ^7^Key Laboratory for Neuroscience, Ministry of Education/National Health and Family Planning Commission, Peking University, Beijing, China; ^8^Bernal Institute, University of Limerick, Limerick, Ireland; ^9^Health Research Institute (HRI), University of Limerick, Limerick, Ireland

**Keywords:** ASD, autism mouse models, zinc deficiency, SHANK3, brain structures, animal MRI

## Abstract

Research efforts over the past decades have unraveled both genetic and environmental factors, which contribute to the development of autism spectrum disorders (ASD). It is, to date, largely unknown how different underlying causes result in a common phenotype. However, the individual course of development and the different comorbidities might reflect the heterogeneous genetic and non-genetic contributions. Therefore, it is reasonable to identify commonalities and differences in models of these disorders at the different hierarchical levels of brain function, including genetics/environment, cellular/synaptic functions, brain regions, connectivity, and behavior. To that end, we investigated *Shank3* transgenic mouse lines and compared them with a prenatal zinc-deficient (PZD) mouse model of ASD at the level of brain structural alterations in an 11,7 T small animal magnetic resonance imaging (MRI). Animals were measured at 4 and 9 weeks of age. We identified a decreased total brain volume (TBV) and hippocampal size of *Shank3*^−/−^ mice but a convergent increase of basal ganglia (striatum and globus pallidus) in most mouse lines. Moreover, *Shank3* transgenic mice had smaller thalami, whereas PZD mice had this region enlarged. Intriguingly, *Shank3* heterozygous knockout mice mostly showed minor abnormalities to full knockouts, which might reflect the importance of proper *Shank3* dosage in neuronal cells. Most reported volume changes seemed to be more pronounced at younger age. Our results indicate both convergent and divergent brain region abnormalities in genetic and non-genetic models of ASD. These alterations of brain structures might be mirrored in the reported behavior of both models, which have not been assessed in this study.

## Introduction

Autism spectrum disorders (ASD) belong to the most common neurodevelopmental disorders with a prevalence of approximately 1% in the population. Affected individuals demonstrate as consensus criteria abnormal social behavior and communication as well as repetitive behavior. However, a majority also presents a wide variety of comorbidities such as intellectual disability, language impairment, anxiety, hyperactivity, and sensory deficits (Levy et al., 2009).

The underlying pathomechanisms are not yet fully understood, while the causative factors have been unraveled within the last decades. Genetic alterations account for approximately two thirds of all cases (Huguet et al., 2016), and studies on identical twins show high concordance rates with strong penetrance of mutations. We know some of the genetic factors, however, the number of possible mutations is daunting with hundreds of genes potentially contributing to the phenotype (Ellegood, 2012). Nevertheless, there is a large cluster of genes coding for synaptic proteins. In fact, ASD are currently also perceived as synaptopathies (Brose et al., 2010). Mutations in the gene coding for the postsynaptic scaffolding protein SH3 and multiple ankyrin repeat domains 3 (SHANK3) are one of the rather common monogenetic causes of syndromic ASD. The most prominent example of a SHANK3 deficiency is the 22q13.3 deletion syndrome, also known as Phelan-McDermid syndrome, however, intragenic mutations have also been found in autistic cohorts (Grabrucker et al., 2011b). Apart from the strong genetic component in autism, environmental factors and gene-environment interactions are contributing factors. For unknown reasons, every second autistic individual demonstrates zinc deficiency very early in life (Grabrucker, 2012; Grabrucker et al., 2014). Intriguingly, zinc is strongly concentrated in brain tissue and there it effects, among others, the homomerization of SHANK molecules, including SHANK3 (Grabrucker et al., 2011a; Grabrucker, 2014). Maternal zinc deficiency in mice results in ASD-like behavior in the offspring (Grabrucker et al., 2016, 2014).

Neuroanatomical alterations are a frequent finding in many neurological and psychiatric disorders. Kanner (1943) already reported an increased head circumference in 5 of 11 case reports in the initial description of autism. However, head size correlates with brain size only in early childhood but Kanner’s case reports were mostly beyond that period (Kanner, 1943). Approximately 20% of all autistic patients show macrocephaly, at least in a certain period throughout development. Beginning in the 1980ies with computer tomography and further followed with magnetic resonance imaging (MRI), researchers and physicians found a common pattern seen in a large number of affected individuals: the brain size at birth is usually normal, while an accelerated growth can be observed in early childhood (up to 4 years), which affects up to 90%. Then, a deceleration follows with a plateau phase until normal volume values are reached again by entry into adolescence or early adulthood (Chen et al., 2011; Stigler et al., 2011; Zielinski et al., 2014). The detailed volumetric deviation of specific parts of the brain is highly heterogeneous, which is attributable to age, intelligence quotient, but predominantly to the underlying genetic alterations. To reduce this factor, it has been reasoned to analyze neuroanatomy of syndromal ASD in humans and to model the different disease entities in mouse models, which allow for a specific genotype-phenotype correlation (Ellegood, 2012).

Nieman et al. (2007) analyzed mouse models mimicking a variety of different neurological diseases and, thereby, observed detectable changes in 90% of them. A proper model for a neuropsychiatric disease such as autism harbors the same mutations as in humans, demonstrates comparable phenotypes, and, finally, also the same underlying molecular, cellular, or neuroanatomical changes, which are ideally reproducible (Ellegood and Crawley, 2015). To date, several autism mouse models have been analyzed on the level of neuroanatomical changes with MRI. These studies included mutations in synaptic genes (Ellegood et al., 2010, 2011, 2012, 2015a; Peça et al., 2011; Kumar et al., 2014; Steadman et al., 2014), CNVs (copy number variations; Horev et al., 2011; Ellegood et al., 2015a,b), and inbred mouse strains including the BTBR mouse with autistic-like behavior (Ellegood et al., 2013, 2015a). An important conclusion is that the commonness of neuroanatomical alterations is even higher as compared to humans and that the heterogeneity is as high as observed in autistic individuals. However, the reproducibility of findings in the same model seems to be remarkably high (Ellegood and Crawley, 2015).

Along this line, we aimed at adding a longitudinal study with measurement points in: (1) the juvenile age; and (2) early adulthood by comparing a genetic model with an environmental model of autism, which have already been extensively characterized by our workgroups. The transgenic *Shank3* isoform-specific knockout mice have not been assessed on behavioral level by us (Schmeisser et al., 2012), but similar models show social abnormalities, reduced vocalization, and repetitive behavior (Bozdagi et al., 2010; Peça et al., 2011; Wang et al., 2011; Bozdagi et al., 2013). Non-genetic autism models have hardly been described but Grabrucker et al. (2014) introduced a model of prenatal zinc deficiency (PZD). Female mice undergo a temporary nutritional deprivation from zinc before and during pregnancy. The PZD offspring demonstrates autistic-like behavior. Intriguingly, one feature of these mice is a synaptic downregulation of SHANK3 levels that recovers only after birth with adequate zinc supply, and a loss of excitatory synapses in several brain regions (Grabrucker et al., 2014). Therefore, PZD and transgenic *Shank3* mice have a shared feature on a molecular level with a functional relationship at synapses. Our hypothesis is that it is possible to gain further insights in the neurobiology underlying the ASD phenotypes by further analyzing the neuroanatomy with MRI of two different models of ASD, one produced by a genetic and one by a non-genetic factor. It is plausible to assume: (1) convergent neuroanatomical changes associated with common autistic phenotypes; and (2) divergent findings may relate to specific comorbidities present in only one model.

## Materials and Methods

### Animal Ethics Statement

All animal experiments were performed in compliance with the guidelines for the welfare of experimental animals issued by the Federal Government of Germany, the National Institutes of Health and the Max Planck Society. The experiments in this study were approved by the review board of the Land Baden-Württemberg (Regierungspräsidium Tübingen) and the local ethics committee at Ulm University, permit number 1239.

### Animal Breeding

All animals were bred and mated in the animal facility of Ulm University.

#### *Shank3* Transgenic Mice

*Prosap2*/*Shank3* mutants were generated by Genoway (Lyon, France) on a C57BL/6 strain background. The targeting strategy of the isoform-specific knockout has been described from our laboratory by Schmeisser et al. (2012). In synopsis, exon 11 in the SH3 domain was deleted, thereby, resulting in a translational stop sequence. The western blot phenotype with missing α- and β-isoforms was referred to the genotype *Prosap2/Shank3αβ*^−/−^ (Schmeisser et al., 2012). The strains are, further on, named as *Shank3*^+/–^ for heterozygous and *Shank3*^−/−^ for homozygous animals.

#### Prenatal Zinc-Deficient Mice

PZD mice were generated as described previously (Grabrucker et al., 2014, 2016) using C57BL/6 mice purchased from Janvier Labs. In brief, 8-week-old mice were purchased from Janvier Labs and housed in plastic cages under standard laboratory conditions [22°C, 12 h rhythm (lights on at 7 am)], and provided with food and water available *ad libitum*. After 1 week of acclimation, mice were divided into two groups; one group was fed a zinc-deficient diet [4 parts per million (ppm; zinc, SSNIFF diets, Germany)] with demineralized drinking water, whereas the control group was fed with standard laboratory food (35 ppm zinc). After 5 weeks, females of the control and zinc-deficient group were mated and maintained on their respective diet during pregnancy. To prevent zinc contamination, feeding jars, water bottles, and plastic cages were rinsed with HCl and deionized water. After birth, offspring from both control and PZD mice received milk from mice on standard laboratory diet and were fed standard laboratory food after weaning.

### Small Animal MRI

The MRI measurements were performed in the small animal MRI of Ulm University.

#### Animal Narcosis

High-resolution MRI experiments on age-matched control, PZD, and *Shank3* transgenic mice were carried out under isoflurane anesthesia (5% for induction, 1.5% for maintenance, mixed with air).

#### Structural MRI Scans

All data were acquired on a dedicated small bore animal scanner (Biospec 117/16, Bruker, Ettlingen, Germany) equipped with a cryogenically cooled two-element surface (MRI CryoProbeTM, Bruker BioSpec, Ettlingen, Germany) transmit/receive coil. Anatomical brain images were acquired in axial, sagittal, and coronal slice orientation applying a gradient-echo (FLASH) sequence with acquisition parameters as: TE/TR 2.2/193 ms (TE, echo time; TR, repetition time), matrix 260 × 260, Δ*r* = 65 × 65 × 500 μm^3^.

In order to not overburden the mice, all measurements together were at an upper limit of narcosis time, thus, the resolution of the scans had to be restricted, e.g., to 500 μm slice thickness for the FLASH sequence [similar resolution as already was used in (Braunstein et al., 2010; Wiesner et al., 2015)]. Thus, we focussed especially on those brain regions, which: (1) are clearly distinguishable; (2) have a certain extension mediolaterally (for sagittal plane analyses) or rostrocaudally (for coronal plane analyses); and (3) have a supposedly high relevance in autism development.

### Data Analysis

Volumetric tissue analysis followed previously established semi-automatic procedures in (Wiesner et al., 2015; Braunstein et al., 2010): data processing was performed by the in-house developed software package Tissue Classification Software (TCS). For optimized visualization, the acquisition matrix of 260 × 260 voxels was transformed into a 768 × 768 grid by nearest neighbor interpolation. Slicewise filtering was applied to equalize intensity gradients caused by recording. TCS includes mouse-based drawing tools for tissue/voxel selection. In order to define clearly visible regions, drawing was supported by a two-level thresholded conventional region-grow algorithm. Following the operator-defined intensity threshold, all connected voxels with respect to their intensity within the predefined intensity range were selected (process described in Supplementary Figure S1). The analysis was blinded and evaluated by the same experienced investigator. All regions were checked again by several investigators.

Brain regions and bregma coordinates were identified manually according to Allen Brain Atlas (Allen Institute for Brain Science, 2011) and Franklin and Paxinos (2007). In the following, brain region delineation is described in more detail. Total brain volume (TBV) and cerebellum were analyzed in sagittal planes. In order to separate left and right hemispheres, the midsagittal section volume was assigned half to both volumes each. Brain-spinal cord transition was determined as a tangential line at the posterior end of the cerebellum. All other brain areas were analyzed in coronal planes. In the following, we refer to the analyzed areas as they are named in the Allen Brain Atlas. Hippocampus was analyzed as denominated hippocampal region and subiculum under the hippocampal formation structures. Striatum was analyzed as denominated caudoputamen. Globus pallidus was analyzed as denominated globus pallidus, external and internal segment in the atlas. Thalamus was analyzed as denominated thalamus.

TBV and striatum in PZD mice were already analyzed by Grabrucker et al. (2018) however, with a different anaylsis tool, which resulted in slightly different anatomical boundaries.

Cortical thickness was measured at the bregma coordinates −0.82 mm, −1.28 mm, and −2.75 mm (adult mouse brain atlas of the Allen Brain Atlas) for both hemispheres. Thereby, a perpendicular line was drawn at the most dorsal extension of the corpus callosum. Brains at 4 and 9 weeks were not obviously different at the mentioned bregma coordinates, which is reflected in the almost identical TBV.

Statistical analysis was performed with GraphPad Prism 5 for Supplementary Figures and 7 for main figures (GraphPad Software, La Jolla, USA).

Significances were calculated with a two-way analysis of variance (ANOVA) with a Bonferroni *post hoc* test. Significance levels are as follows according to the *p*-value threshold: *p < 0.05, **p < 0.01, ***p < 0.001.

## Results

### Study Concept

**Figure 1:**
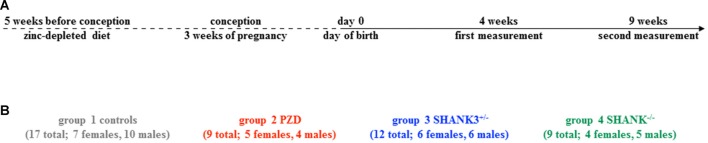
Study design with autism mouse models measured at adolescent and adult ages. **(A)** The timeline of this longitudinal study shows the two measurement points at 4 weeks and 9 weeks. **(B)** In total, 17 controls (7 females, 10 males), nine PZD (5 females, 4 males), 12 *Shank3*^+/–^ (6 females, 6 males), and nine *Shank3*^−/−^ (4 females, 5 males) mice were measured.

The study was conceptualized longitudinally with horizontal measurements at two different ages: postnatal day (PD) 28–30 (4 weeks) and PD 63–65 (9 weeks; Figure 1A). This interval spans the time corresponding to human adolescence and early adulthood, respectively. Per group, two different cohorts were measured and the measurement regimen for both ages was kept unchanged during the entire study. Three different autism mouse models were analyzed, namely a heterozygous and a full knockout of *Shank3* isoforms (*Shank3*^+/–^ and *Shank3*^−/−^), and a model of PZD (Figure 1B). All shown data points including mean values, standard error of the mean, standard deviation, and *p*-values of the main figures are provided in Supplementary Table S1.

### Analysis of Total Brain Volume and Cortical Brain Regions

**Figure 2:**
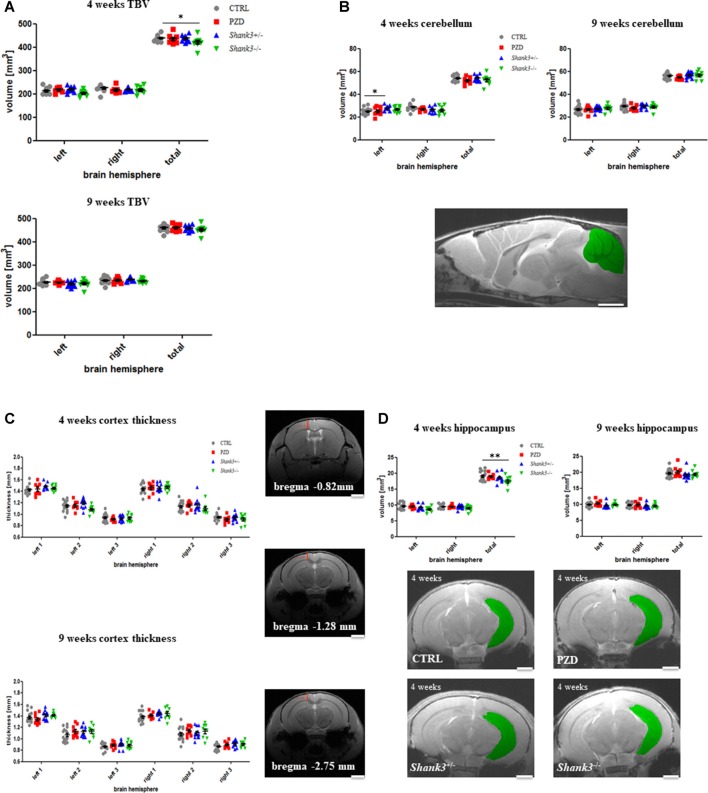
Brain region volumetry of the total brain and cortical structures. Volumetric and thickness analyses are provided for the total brain **(A)**, the cerebellum **(B)**, cortical thickness **(C)**, and hippocampus **(D)**. Exemplary images are displayed of a 4-weeks-old control line mouse as a mid-sagittal section with the cerebellum in green **(B)**, at the three bregma coordinates of a control mouse in coronal sections **(C)**, and as 2D images in coronal sections at 4 weeks for the hippocampus measurements **(D)**. The hippocampus is highlighted in green. CTRL, controls; L, left; PZD, prenatal zinc-deficient mice; R, right; *Shank3*, SH3 and multiple ankyrin repeat domains 3; TBV, total brain volume. The mean values in the diagrams are presented with standard errors of the mean. Significance levels are as follows according to the *p*-value threshold: < 0.05 = *, < 0.01 = **. Scale bars represent 2 mm.

The analysis encompassed the TBV, the cerebellar volume, and the following cortical areas: cortical thickness at three corresponding points at the bregma coordinates −0.82 mm, −1.28 mm, and −2.75 mm (no volume measured), and hippocampus. All measurements were conducted on right and left parts of the brain (Figure 2).

Initially, the TBV was investigated (Figure 2A). The comparison also included right vs. left hemispheres of the brain. The only significant finding was in *Shank3*^−/−^ mice at 4 weeks of age, a trend is seen in both hemispheres at this age, however, not significant. A trend can still be observed for this strain at 9 weeks.

The cerebellar volume was not majorly different between the cohorts (Figure 2B). However, there was an increase of the left hemisphere in *Shank3*^+/–^. This alteration seemed to vanish over time.

Next, the cortical thickness was determined at three different bregma coordinates (Figure 2C). No significant alterations were detected. In some of the autism mouse models at 9 weeks, a slight tendency of increased thickness could be observed.

Finally, we were interested in the volume of the archicortex, namely the hippocampus (Figure 2D). Here, the *Shank3*^−/−^ mice showed a marked decrease in volume at the early age, which seemed to largely disappear later. These results are attributable to changes in both hemispheres, however, not significant. A non-significant trend can also be seen in the heterozygous *Shank3*^+/–^ animals.

### Analysis of Subcortical Brain Regions

**Figure 3:**
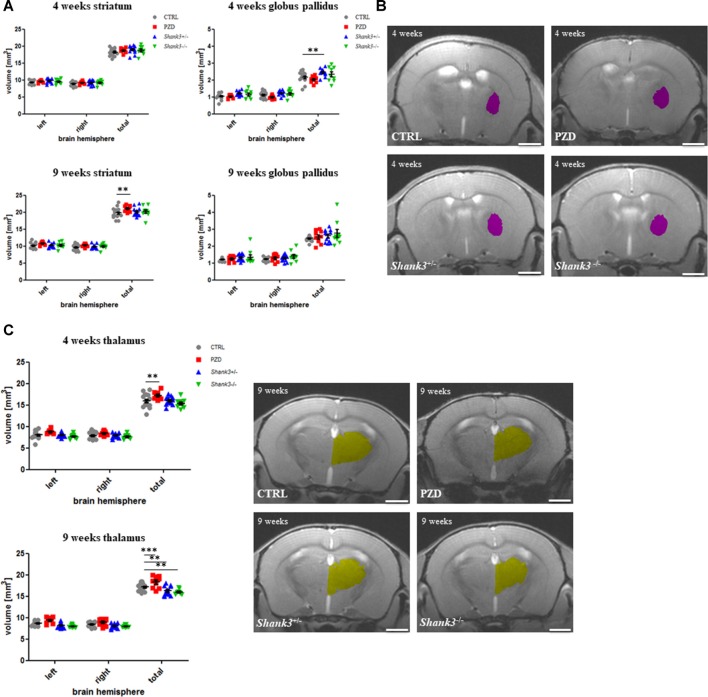
Brain region volumetry of subcortical areas. The volumetric results of the subcortical nuclei are shown for the striatum **(A)**, globus pallidus **(B)**, and thalamus **(C)**. Exemplary images of coronal sections of 4-weeks-old mice are depicted for globus pallidus (violet, **B**) and of 9-week-old mice for thalamus (yellow, **C**). CTRL, controls; L, left; PZD, prenatal zinc-deficient mice; R, right; *Shank3*, SH3 and multiple ankyrin repeat domains 3. The mean values in the diagrams are presented with standard errors of the mean. Significance levels are as follows according to the *p*-value threshold: < 0.01 = **, < 0.001 = ***. Scale bars represent 2 mm.

In Figure 3, the analysis for size differences for the subcortical regions striatum, globus pallidus, and thalamus are displayed. The striatum was tendentially bigger in all autism mouse models at both ages, however, only significant in the PZD mice at 9 weeks (Figure 3A).

The globus pallidus volumetry revealed significant differences in the *Shank3* mouse models (Figure 3B). The added hemisphere volumes of this structure were increased in volume at both ages, however, significant only for *Shank3*^+/–^ at 4 weeks.

Lastly, the thalamus measurements revealed a reduction of volume in both *Shank3* models, which was only significant for the late measurement (Figure 3C). Interestingly, the PZD mice showed, in turn, an increase of the thalamic volume on both sides and throughout development. These differences seemed to be attributable to both hemispheres.

### Region Volumetry Relative to TBV and Sex Differences

Finally, we aimed at investigating, in how the brain region volumes depend on the respective TBV. Results were not majorly different, however, some signficicancies hold, others disapper (see Supplementary Figure S2).

Although most significant results disappear because of low individual male and female numbers, we found it interesting to split the groups according to sex (Supplementary Figure S3). Interestingly, when significances remained, they usually could be attributed to the male mice.

## Discussion

### Main Findings

In a longitudinal study, we compared brain anatomical alterations with small animal MRI in two different mouse models of ASD, a non-genetic, and a *Shank3* transgenic model with two different gene dosages. We have, to our knowledge, performed the first study analyzing a non-genetic autism mouse model in comparison to a genetic model in order to investigate the shared neurobiological substrate of their common ASD behavioral phenotype. The study revealed the following main findings:

1. All ASD models in this study show largely unaltered cerebellar volumes, cortical thickness, however, TBV and hippocampus are diminished in *Shank3* full knockout mice.
2. A convergent finding is an increased size of basal ganglia structures; striatum is significantly enlarged in PZD mice as was *Shank3* mice globus pallidus in all models.
3. *Shank3* models additionally show a decrease in the thalamus volume, on the contrary, PZD mice had this area enlarged.
4. Both heterozygous and full *Shank3* isoform-specific knockout mice display gene-dosage dependent alterations with more pronounced changes in full knockout animals.
5. Male mice show more significant alterations.
6. Some of the alterations are transient, with early brain development being more divergent from control mice.

### *Shank3* and PZD Mice Demonstrate Convergent Basal Ganglia but Divergent Total Brain Volume, Thalamic and Hippocampal Alterations

In the following, the results of this study will be discussed with regard to previous investigations for neuroanatomical alterations in mouse models and comparable observations in humans. The focus will, further on, be set on both similarities and discrepant results.

#### Brain Structures in Mouse Models for ASD

To date, most analyzed autism mouse models have not been studied longitudinally as in our study. In the majority of cases, MRI scans represented endpoints of the studies with *post mortem* MRI. Only in one previous work the developmental stages are comparable to our early measurements at about 4 weeks of age, resembling the human juvenile age, (Ellegood et al., 2010), whereas most measurements were conducted at an age around our second time-point, which corresponds to early adulthood (Ellegood et al., 2012, 2013, 2015a,b). Only Kumar et al. (2014) conducted a longitudinal study with three consecutive measurements starting at PD 30 followed by two more runs with two 20 days intervals. They report that the neuroanatomical changes are developmentally stable (Kumar et al., 2014). Along these lines, our results with regard to dynamic over time are important information, as not all anatomical alterations were maintained during development. For example, the changes in the TBV, the subtle changes in hemispheric cerebellar, hippocampal, and globus pallidus volumes of *Shank3* models were more pronounced at younger age. The alterations of brain structures in the non-genetic PZD model seemed to be more stable over time, e.g., in the thalamus. We want to emphasize that demarcation of the respective brain regions might be subtly different in the conducted studies. We strictly followed anatomical descriptions of the Allen Brain Atlas (Allen Institute for Brain Science, 2011).

Changes in the volume of basal ganglia (especially striatum) of autism mouse models have repeatedly been reported. But most models show a decrease, such as models with a *Nlgn3* knock-in (Ellegood et al., 2011; Kumar et al., 2014), 15q11–13 duplication (Ellegood et al., 2015a), an *Fmr1* knockout (Ellegood et al., 2010), an *Itgβ3* model (Ellegood et al., 2012), and the BTBR inbred strain (Ellegood et al., 2013). Ellegood and colleagues showed in a cross-sectional study with 26 autism mouse models in 8-week-old mice that only six had an increased striatal size, including a *Shank3* isoform-specific knockout at approx. the same age as our later measurement point (Ellegood et al., 2015a). Interestingly, we could only detect a non-significant enlargement of the striatum and globus pallidus in both *Shank3* models at this age. The slight differences to our study might be due to the different knockout strategy used for the generation of the *Shank3* model; their model had exons 4–9 deleted. Peça et al. (2011) reported an enlarged striatum earlier in the same model. An increased striatal volume in the PZD model was already reported by us earlier with a different analysis method (Grabrucker et al., 2018). It can be concluded that basal ganglia enlargement is a common phenomenon in PZD mice and SHANK3 deficiency, however, it might be dosage-dependent of the remaining isoforms.

Our *Shank3* heterozygous and full knockout mice additionally demonstrated a smaller thalamus as is also observed in a *Nlgn3* knock-in model (Ellegood et al., 2011) and a 15q11–13 duplication model. A comparable situation reported for the striatum is also seen for the thalamus in another study from Ellegood et al. (2015a), showing no alterations in the heterozygous *Shank3* animals but a slight decrease, as seen in our model, in the full knockout. A thalamic enlargement, as seen in the PZD model, is rather rare (Ellegood et al., 2015a). Finally, a change in hippocampus volume is not uncommon in autism mouse models. In line with our study, this archicortical structure is mostly decreased in size including *Shank3* KOs with a different targeting strategy (Ellegood et al., 2011, 2012, 2015a). Also in agreement with Ellegood et al. (2015a), the TBV was reduced in our *Shank3* full KO animals (Ellegood et al., 2015a). In conclusion, although other groups analyzed *Shank3* models with different isoform-specific knockdown strategies, most alterations are in line with previous studies.

Interestingly, when relating the volumes of each brain region to the TBV of the same animals, results were majorly in accordance with the total volume comparison. However, some regions became significantly different or became trends only, e.g., the striatum and the thalamus in *Shank3* transgenic mice, respectively. We recommend to show both total and relative volumes in future studies to unravel or relativize the data.

Moreover, after splitting the data according to sex, we observed more alterations in the male cohorts, which is in line with the male dominance in ASD (Levy et al., 2009). To our knowledge, this is the first study to explicetly report on male-female differences in autism mouse models.

#### Brain Structures in Human Individuals With ASD

Human MRI has a long tradition for diagnosing and monitoring anatomical changes and brain functions in neurological disorders (Anderson and Frank, 2007). Intriguingly, the partly recovery of neuroanatomical changes from adolescence to early adulthood in our study is much in line with what has been observed as a common phenomenon in ASD: an accelerated growth of brain structures during postnatal development with a normalization of most parts until adulthood (Chen et al., 2011; Stigler et al., 2011; Zielinski et al., 2014). Common findings in structural MRI in autistic individuals are an increased TBV, white matter anomalies (including corpus callosum thinning as most frequent neuroanatomical alteration), but also common are alterations of the cerebellum, the hippocampus, the striatum, and the thalamus (Stigler et al., 2011). This is very much in line with our results.

Some models such as a Rett syndrome mouse model have shown quite comparable neuroanatomical alterations as are observed in human patients (Ellegood, 2012). Phelan-McDermid syndrome, which oftentimes goes along with a syndromal variant of ASD, is a SHANK3 deficiency disorder and patients frequently have low zinc levels, which can be a modifying factor of their phenotype (Pfaender et al., 2017). We reasoned that some of the neuroanatomical peculiarities in this syndrome might be replicable in our models. Although several studies have analyzed the brains of affected individuals, the number of included patients was quite low; also the measurements were not always standardized and did not always include thorough volumetric analyses of various brain structures. However, neuroanatomical alterations are abundant in the syndrome including corpus callosum thinning, ventriculomegaly, cortical atrophy, cerebellar malformations, and arachnoid cysts (Aldinger et al., 2013; Soorya et al., 2013; Figura et al., 2014; Philippe et al., 2015). In line with these data, we detected subtle cerebellar changes in one of the *Shank3* mouse models. Future studies ought to focus on precise volumetry of cortical and subcortical structures and would ideally include more cases with isolated *SHANK3* gene disruptions or intragenic mutations to exclude the possibility that other genes than *SHANK3* cause the anatomical alterations. In addition, with reference to the PZD model, it would now be interesting to study a correlation of specific volumetric changes in the subgroup of ASD and Phelan-McDermid syndrome patients with a zinc deficiency as we have seen them in our model organism.

#### Abnormal Brain Structure Associated With Neurobiological and Behavioral Changes in ASD

The following paragraph deals with the question how our observed alterations fit to previous studies on these models. Our *Shank3* model has previously been compared to a *Shank2* full knockout from our lab (Schmeisser et al., 2012). Although behavior was not assessed, similar *Shank3* knockout models display autism-related behavior (Bozdagi et al., 2010; Peça et al., 2011; Wang et al., 2011). Repetitive behavior is the predominant phenotype of these models, which is related to striatal changes (Stigler et al., 2011). This structure is the best analyzed structure in these models and shows most significant alterations with disrupted synaptic assembly and diminished glutamatergic signaling (Peça et al., 2011; Schmeisser et al., 2012). Fitting to our results of volumetric changes in the basal ganglia (striatum and globus pallidus) and thalamus, SHANK3 expression was shown to be predominantly high in these brain regions in comparison to SHANK1 and 2 (Peça et al., 2011).

Also in line with alterations in the striatum, zinc is specifically highly concentrated in this brain region. The PZD model has been shown to display a reduction of excitatory synapses in this area (Grabrucker et al., 2014), where zinc was also shown to interact with SHANK3 at the postsynaptic density. Further, an altered brain lateralization, which among others, affects the dopaminergic transmission in the striatum of these mice was reported (Grabrucker et al., 2018). On behavioral level, this correlates with stereotyped repetitive behaviors such as abnormal circling and altered marble burying that has been reported in PZD mice (Grabrucker et al., 2016), but also other ASD mouse models.

In line with the hippocampus affection that was specific for *Shank3* full KO mice, previous studies report learning and memory deficits (Wang et al., 2011; Yang et al., 2012).

### Conclusions and Outlook

In this study on autism mouse models, SHANK3-deficient mice were compared to PZD mice in terms of neuroanatomical peculiarities measured by structural MRI. While both models display enlarged basal ganglia strucutres, thalami in *Shank3* models were smaller, while they were enlarged in the PZD mice. Especially the latter observation remained significant until early adulthood. Only *Shank3* full knockout mice showed decreased total brain and hippocampal volumes.

Our study harbors several limitations. Because of inter-individual differences, several observed alterations did not turn out be significant or were significant in the early measurement but not in the later analysis. Also, when splitting the group according to sex, we lost some of the significant results. This is most likely attributable to a combination of a low number of individuals and a concomitant low effect size. Future studies might include either more animals or might employ higher resolution. Furthermore, we can only speculate on functional consequences of the observed alterations by reviewing literature on these or comparable models, or the situation in humans. Further, in this study, we cannot answer the question what is happening on the cellular or even molecular levels of the affected areas.

Nevertheless, the results reported here might pave the way for future investigations in order to further scrutinize these observations. This way might lead upstream to unravel the molecular and neurocellular alterations, which could have led to the alterations observed. Moreover, this might give rise to a better understanding of underlying pathomechanisms and to identify therapeutic targets that are shared between the plethora of different causes of ASD. However, based on these data, it is also important to seek downstream to more specifically analyze the models for specific behavioral alterations, which might refer to the brain region alterations in the analyzed models and might be related to specific comorbidities observed in addition to the core features of ASD.

So far, two studies had a glimpse on what is happening on the level of neuronal function and morphology. In an *Fmr1* KO model of autism, two cerebellar nuclei were found to be decreased in size; immunohistochemistry unraveled a loss of neurons accounting for the shrinkage (Ellegood et al., 2010). Peça et al. (2011) nicely showed in *Shank3* transgenic mice, how medium spiny neurons of the striatum are significantly more branched, which goes along with diminished cortico-striatal transmission. In line with our results, the striatum is larger in this model, and the authors suggest these striatal changes to underlie the strong repetitive behavior in this model (Peça et al., 2011). This represents an exemplary study to scrutinize a behavioral alteration on the level of cellular morphology up to the resulting function. Thus, they found a neuroanatomical phenotype on the mesoscopic scale resulting in a distinguishable phenotype. Future investigations might verify these hypotheses of a brain-region—phenotype association by specifical knockdowns of autism-related genes in certain brain regions or key pathways. It could prove the suggested associations between brain regions and a certain phenotype: striatum/globus pallidus—repetitive behavior, hippocampus and thalamus—learning, processing of highly associated sensory input. In line with this, the striatal size in human individuals is negatively correlated with stereotypies but positively with difficulties in problem solving (Stigler et al., 2011). Our data also shows that despite different etiologies of ASDs such as genetic or non-genetic ones, the search of commonalities between different models may identify the neurobiological correlate of the shared behavioral impairments.

Another interesting lesson we learn from our results and by reviewing different mouse models is that it is rather the affection of a certain brain area than the dynamic of increase or decrease in volume, which correlates with a phenotype. This holds true both for human individuals as well as for autism mouse models. An intriguing path would be to correlate the volumetric changes to altered connectivity between brain regions, which has been used to study ASD in both humans as well as mouse models by applying diffusion tensor imaging (DTI).

For our models, it is most intriguing that SHANK3 and zinc have repeatedly been shown to preferentially localize in the same brain areas, to even interact molecularly, and a reduction of both players seems to result in an overlapping phenotype (Bozdagi et al., 2010; Peça et al., 2011; Schmeisser et al., 2012; Grabrucker et al., 2014). However, on the level of neuroanatomical alterations they show similarities but also differ in some respect. This might indicate that the zinc-SHANK3 interaction may be a major driver for the ASD-associated behavioral impairments seen in PZD mice, but also hints toward other SHANK3-independent functions of zinc signaling in the brain. The shared but also unique functions of zinc and possibly a variety of genetic and non-genetic factors may account for the phenotypic heterogeneity of ASD despite the occurrence of common core features. However, it is possible that the molecular and cellular changes might be very similar despite differences in volume changes of certain brain regions. For instance, both *Shank3* and PZD models demonstrate decreased glutamatergic transmission in both the striatum and hippocampus (Bozdagi et al., 2010; Peça et al., 2011; Schmeisser et al., 2012; Grabrucker et al., 2014), but effects on hippocampal volume were only seen in *Shank3* mice.

Intriguingly, our analyzed heterozygous *Shank3* KO was generally less affected than the full KO. This should be further scrutinized on other levels, cellular and behavioral ones, since heterozygous *Shank3* KO more closely resembles the human pathology on the genetic level. Furthermore, it would be worth to study the affection of the thalamus in *Shank3* models in more detail. A common observation in patients with SHANK3 deficiency (Soorya et al., 2013) and mouse models is an altered perception of inconvenient sensations such as heat or pain. If this is only attributable to peripheral neurons as suggested by Han et al. (2016) or also includes higher brain function with sensory processing in the thalamus remains elusive. Moreover, future studies on human individuals with either SHANK3 loss or early life exposure to zinc deficiency might now seek for similar alterations as we have seen them in the corresponding animal models.

Taken together, efforts in describing the brain anatomy in ASD and respective models might be important to: (1) find diagnostic criteria based on MRI-detectable changes (Stevenson and Kellett, 2010); and to (2) pinpoint clusters of ASD variants with same anatomical alterations, maybe associated with similar symptoms constellations, which might then be grouped and subjected to a more specific symptom-based therapy. Intriguingly, such a therapeutic intervention and the putative positive effect might even be measurable as was shown for a Rett model, in which alterations in brain structures was used as marker. After exposure to an enriched environment, some of the neuroanatomical alterations could be rescued (Nag et al., 2009).

## Author Contributions

TB, MS, JS, AA, AG, RZ, TS, and VR designed and outlined this study. HA, AA, MS, and AS carried out all experiments. JB took care of animal breeding. HA, HB, H-PM, JK, and MS jointly analyzed the data. HA, HB, and MS composed the figures. MS, TB, AG, H-PM, and JK jointly wrote the manuscript.

## Conflict of Interest Statement

The authors declare that the research was conducted in the absence of any commercial or financial relationships that could be construed as a potential conflict of interest. The Reviewer EK is currently editing a Research Topic with one of the authors TB, and confirms the absence of any other collaboration.

## Supplementary Material

FIGURE S1 Volumetric analysis cascade. Original data were optionally gradient filtered and an intensity thresholding supported the user-defined selection of the respective brain region. Volumetric marked regions were then analyzed for the respective slices.

FIGURE S2 Volumes relative to TBV. Each measured brain region (not the cortical thickness) was calculated as the percentage of the total brain volume of the same animal. CTRL, controls; L, left; PZD, prenatal zinc-deficient mice; R, right; *Shank3*, SH3 and multiple ankyrin repeat domains 3; TBV, total brain volume. The mean values in the diagrams are presented with standard errors of the mean. Significance levels are as follows according to the *p*-value threshold: < 0.05 = *, < 0.01 = **, < 0.001 = ***.

FIGURE S3 Sex differences. Animals were split according to sex. CTRL, controls; f, female; L, left; m, male; PZD, prenatal zinc-deficient mice; R, right; *Shank3*, SH3 and multiple ankyrin repeat domains 3; TBV, total brain volume. The mean values in the diagrams are presented with standard errors of the mean. Significance levels are as follows according to the *p*-value threshold: < 0.05 = *, < 0.01 = **, < 0.001 = ***.

TABLE S1 Raw volume data and statistics for Figures 2, 3.

## Abbreviations

ASD: autism spectrum disorder(s)
MRI: magnetic resonance imaging
ppm: parts per million
PZD: prenatal zinc-deficient
SHANK: SH3 and multiple ankyrin repeat domains
TBV: total brain volume
TCS: Tissue Classification Software.

## References

[B1] Aldinger K. A. Kogan J. Kimonis V. Fernandez B. Horn D. Klopocki E.. (2013). Cerebellar and posterior fossa malformations in patients with autism-associated chromosome 22q13 terminal deletion. Am. J. Med. Genet. A 161A, 131–136. 10.1002/ajmg.a.35700 23225497 PMC3733662

[B100] Allen Institute for Brain Science (2011). Allen Brain Atlas API. Available online at: http://atlas.brain-map.org/

[B2] Anderson S. A. Frank J. A. (2007). MRI of mouse models of neurological disorders. NMR Biomed. 20, 200–215. 10.1002/nbm.1167 17451184

[B3] Bozdagi O. Sakurai T. Papapetrou D. Wang X. Dickstein D. L. Takahashi N.. (2010). Haploinsufficiency of the autism-associated Shank3 gene leads to deficits in synaptic function, social interaction, and social communication. Mol. Autism 1:15. 10.1186/2040-2392-1-15 21167025 PMC3019144

[B4] Bozdagi O. Tavassoli T. Buxbaum J. D. (2013). Insulin-like growth factor-1 rescues synaptic and motor deficits in a mouse model of autism and developmental delay. Mol. Autism 4:9. 10.1186/2040-2392-4-9 23621888 PMC3649942

[B5] Braunstein K. E. Eschbach J. Ròna-Vörös K. Soylu R. Mikrouli E. Larmet Y.. (2010). A point mutation in the dynein heavy chain gene leads to striatal atrophy and compromises neurite outgrowth of striatal neurons. Hum. Mol. Genet. 19, 4385–4398. 10.1093/hmg/ddq361 20807776 PMC3298848

[B6] Brose N. O’Connor V. Skehel P. (2010). Synaptopathy: dysfunction of synaptic function? Biochem. Soc. Trans. 38, 443–444. 10.1042/bst0380443 20298199

[B7] Chen R. Jiao Y. Herskovits E. H. (2011). Structural MRI in autism spectrum disorder. Pediatr. Res. 69, 63R–68R. 10.1203/pdr.0b013e318212c2b3 21289538 PMC3081653

[B15] Ellegood J. (2012). Magnetic resonance imaging as a tool for the study of mouse models of autism. Autism 01:008 10.4172/2165-7890.s1-008

[B8] Ellegood J. Anagnostou E. Babineau B. A. Crawley J. N. Lin L. Genestine M.. (2015a). Clustering autism: using neuroanatomical differences in 26 mouse models to gain insight into the heterogeneity. Mol. Psychiatry 20, 118–125. 10.1038/mp.2014.98 25199916 PMC4426202

[B13] Ellegood J. Nakai N. Nakatani J. Henkelman M. Takumi T. Lerch J. (2015b). Neuroanatomical phenotypes are consistent with autism-like behavioral phenotypes in the 15q11–13 duplication mouse model. Autism Res. 8, 545–555. 10.1002/aur.1469 25755142

[B10] Ellegood J. Babineau B. A. Henkelman R. M. Lerch J. P. Crawley J. N. (2013). Neuroanatomical analysis of the BTBR mouse model of autism using magnetic resonance imaging and diffusion tensor imaging. Neuroimage 70, 288–300. 10.1016/j.neuroimage.2012.12.029 23275046 PMC3595420

[B9] Ellegood J. Crawley J. N. (2015). Behavioral and neuroanatomical phenotypes in mouse models of autism. Neurotherapeutics 12, 521–533. 10.1007/s13311-015-0360-z 26036957 PMC4489953

[B14] Ellegood J. Henkelman R. M. Lerch J. P. (2012). Neuroanatomical assessment of the integrin β3 mouse model related to autism and the serotonin system using high resolution MRI. Front. Psychiatry 3:37. 10.3389/fpsyt.2012.00037 22557981 PMC3337465

[B11] Ellegood J. Lerch J. P. Henkelman R. M. (2011). Brain abnormalities in a neuroligin3 R451C knockin mouse model associated with autism. Autism Res. 4, 368–376. 10.1002/aur.215 21882360

[B12] Ellegood J. Pacey L. K. Hampson D. R. Lerch J. P. Henkelman R. M. (2010). Anatomical phenotyping in a mouse model of fragile X syndrome with magnetic resonance imaging. Neuroimage 53, 1023–1029. 10.1016/j.neuroimage.2010.03.038 20304074

[B16] Figura M. G. Coppola A. Bottitta M. Calabrese G. Grillo L. Luciano D.. (2014). Seizures and EEG pattern in the 22q13.3 deletion syndrome: clinical report of six italian cases. Seizure 23, 774–779. 10.1016/j.seizure.2014.06.008 25027555

[B330] Franklin K. B. J. Paxinos G. (2007). The Mouse Brain in Stereotaxic Coordinates. 3rd Edn. San Diego, CA: Academic Press.

[B17] Grabrucker A. M. Knight M. J. Proepper C. Bockmann J. Joubert M. Rowan M.. (2011a). Concerted action of zinc and ProSAP/Shank in synaptogenesis and synapse maturation. EMBO J. 30, 569–581. 10.1038/emboj.2010.336 21217644 PMC3034012

[B20] Grabrucker A. M. Schmeisser M. J. Schoen M. Boeckers T. M. (2011b). Postsynaptic ProSAP/Shank scaffolds in the cross-hair of synaptopathies. Trends Cell Biol. 21, 594–603. 10.1016/j.tcb.2011.07.003 21840719

[B18] Grabrucker A. M. (2012). Environmental factors in autism. Front. Psychiatry 3:118. 10.3389/fpsyt.2012.00118 23346059 PMC3548163

[B19] Grabrucker A. M. (2014). A role for synaptic zinc in ProSAP/Shank PSD scaffold malformation in autism spectrum disorders. Dev. Neurobiol. 74, 136–146. 10.1002/dneu.22089 23650259 PMC4272576

[B23] Grabrucker S. Boeckers T. M. Grabrucker A. M. (2016). Gender dependent evaluation of autism like behavior in mice exposed to prenatal zinc deficiency. Front. Behav. Neurosci. 10:37. 10.3389/fnbeh.2016.00037 26973485 PMC4776245

[B21] Grabrucker S. Haderspeck J. C. Sauer A. K. Kittelberger N. Asoglu H. Abaei A.. (2018). Brain lateralization in mice is associated with zinc signaling and altered in prenatal zinc deficient mice that display features of autism spectrum disorder. Front. Mol. Neurosci. 10:450. 10.3389/fnmol.2017.00450 29379414 PMC5775238

[B22] Grabrucker S. Jannetti L. Eckert M. Gaub S. Chhabra R. Pfaender S.. (2014). Zinc deficiency dysregulates the synaptic ProSAP/Shank scaffold and might contribute to autism spectrum disorders. Brain 137, 137–152. 10.1093/brain/awt303 24277719

[B24] Han Q. Kim Y. H. Wang X. Liu D. Zhang Z. J. Bey A. L.. (2016). SHANK3 deficiency impairs heat hyperalgesia and TRPV1 signaling in primary sensory neurons. Neuron 92, 1279–1293. 10.1016/j.neuron.2016.11.007 27916453 PMC5182147

[B25] Horev G. Ellegood J. Lerch J. P. Son Y.-E. Muthuswamy L. Vogel H.. (2011). Dosage-dependent phenotypes in models of 16p11.2 lesions found in autism. Proc. Natl. Acad. Sci. U S A 108, 17076–17081. 10.1073/pnas.1114042108 21969575 PMC3193230

[B26] Huguet G. Benabou M. Bourgeron T. (2016). The Genetics of Autism Spectrum Disorders. Edited by Paolo Sassone-Corsi and Yves Christen. A Time for Metabolism and Hormones. Research and Perspectives in Endocrine Interactions. Cham: Springer International Publishing. 28892342

[B27] Kanner L. (1943). Autistic disturbance of affective contact. Nervous Child 2, 217–250.

[B28] Kumar M. Duda J. T. Hwang W.-T. Kenworthy C. Ittyerah R. Pickup S.. (2014). High resolution magnetic resonance imaging for characterization of the neuroligin-3 knock-in mouse model associated with autism spectrum disorder. PLoS One 9:e109872. 10.1371/journal.pone.0109872 25299583 PMC4192590

[B29] Levy S. E. Mandell D. S. Schultz R. T. (2009). Autism. Lancet 374, 1627–1638. 10.1016/S0140-6736(09)61376-3 19819542 PMC2863325

[B30] Nag N. Moriuchi J. M. Peitzman C. G. Ward B. C. Kolodny N. H. Berger-Sweeney J. E. (2009). Environmental enrichment alters locomotor behaviour and ventricular volume in Mecp2 1lox mice. Behav. Brain Res. 196, 44–48. 10.1016/j.bbr.2008.07.008 18687363

[B31] Nieman B. J. Lerch J. P. Bock N. A. Chen X. J. Sled J. G. Henkelman R. M. (2007). Mouse behavioral mutants have neuroimaging abnormalities. Hum. Brain Mapp. 28, 567–575. 10.1002/hbm.20408 17437292 PMC6871448

[B32] Peça J. Feliciano C. Ting J. T. Wang W. Wells M. F. Venkatraman T. N.. (2011). *Shank3* mutant mice display autistic-like behaviours and striatal dysfunction. Nature 472, 437–442. 10.1038/nature09965 21423165 PMC3090611

[B33] Pfaender S. Sauer A. K. Hagmeyer S. Mangus K. Linta L. Liebau S.. (2017). Zinc deficiency and low enterocyte zinc transporter expression in human patients with autism related mutations in SHANK3. Sci. Rep. 7:45190. 10.1038/srep45190 28345660 PMC5366950

[B34] Philippe A. Craus Y. Rio M. Bahi-Buisson N. Boddaert N. Malan V.. (2015). Case report: an unexpected link between partial deletion of the *SHANK3* gene and heller’s *dementia infantilis*, a rare subtype of autism spectrum disorder. BMC Psychiatry 15:256. 10.1186/s12888-015-0631-6 26489495 PMC4618364

[B35] Schmeisser M. J. Ey E. Wegener S. Bockmann J. Stempel A. V. Kuebler A.. (2012). Autistic-like behaviours and hyperactivity in mice lacking ProSAP1/Shank2. Nature 486, 256–260. 10.1038/nature11015 22699619

[B36] Soorya L. Kolevzon A. Zweifach J. Lim T. Dobry Y. Schwartz L.. (2013). Prospective investigation of autism and genotype-phenotype correlations in 22q13 deletion syndrome and *SHANK3* deficiency. Mol. Autism 4:18. 10.1186/2040-2392-4-18 23758760 PMC3707861

[B37] Steadman P. E. Ellegood J. Szulc K. U. Turnbull D. H. Joyner A. L. Henkelman R. M.. (2014). Genetic effects on cerebellar structure across mouse models of autism using a magnetic resonance imaging atlas. Autism Res. 7, 124–137. 10.1002/aur.1344 24151012 PMC4418792

[B38] Stevenson J. L. Kellett K. A. (2010). Can magnetic resonance imaging aid diagnosis of the autism spectrum? J. Neurosci. 30, 16763–16765. 10.1523/JNEUROSCI.4946-10.2010 21159947 PMC6634910

[B39] Stigler K. A. McDonald B. C. Anand A. Saykin A. J. McDougle C. J. (2011). Structural and functional magnetic resonance imaging of autism spectrum disorders. Brain Res. 1380, 146–161. 10.1016/j.brainres.2010.11.076 21130750 PMC3465665

[B40] Wang X. McCoy P. A. Rodriguiz R. M. Pan Y. Je Shawn H. Roberts A. C.. (2011). Synaptic dysfunction and abnormal behaviors in mice lacking major isoforms of Shank3. Hum. Mol. Genet. 20, 3093–3108. 10.1093/hmg/ddr212 21558424 PMC3131048

[B41] Wiesner D. Sinniger J. Henriques A. Dieterlé S. Müller H.-P. Rasche V.. (2015). Low dietary protein content alleviates motor symptoms in mice with mutant dynactin/dynein-Mediated neurodegeneration. Hum. Mol. Genet. 24, 2228–2240. 10.1093/hmg/ddu741 25552654 PMC4447824

[B42] Yang M. Bozdagi O. Scattoni M. L. Wöhr M. Roullet F. I. Katz A. M.. (2012). Reduced excitatory neurotransmission and mild autism-relevant phenotypes in adolescent Shank3 null mutant mice. J. Neurosci. 32, 6525–6541. 10.1523/JNEUROSCI.6107-11.2012 22573675 PMC3362928

[B43] Zielinski B. A. Prigge M. B. D. Nielsen J. A. Froehlich A. L. Abildskov T. J. Anderson J. S.. (2014). Longitudinal changes in cortical thickness in autism and typical development. Brain 137, 1799–1812. 10.1093/brain/awu083 24755274 PMC4032101

