# Supplementary figures and images for: Shank3 Transgenic and Prenatal Zinc-Deficient Autism Mouse Models Show Convergent and Individual Alterations of Brain Structures in MRI
Source: Front Neural Circuits. 2019 Feb 22;13:6. doi: 10.3389/fncir.2019.00006 (PMC6395436; doi:10.3389/fncir.2019.00006)

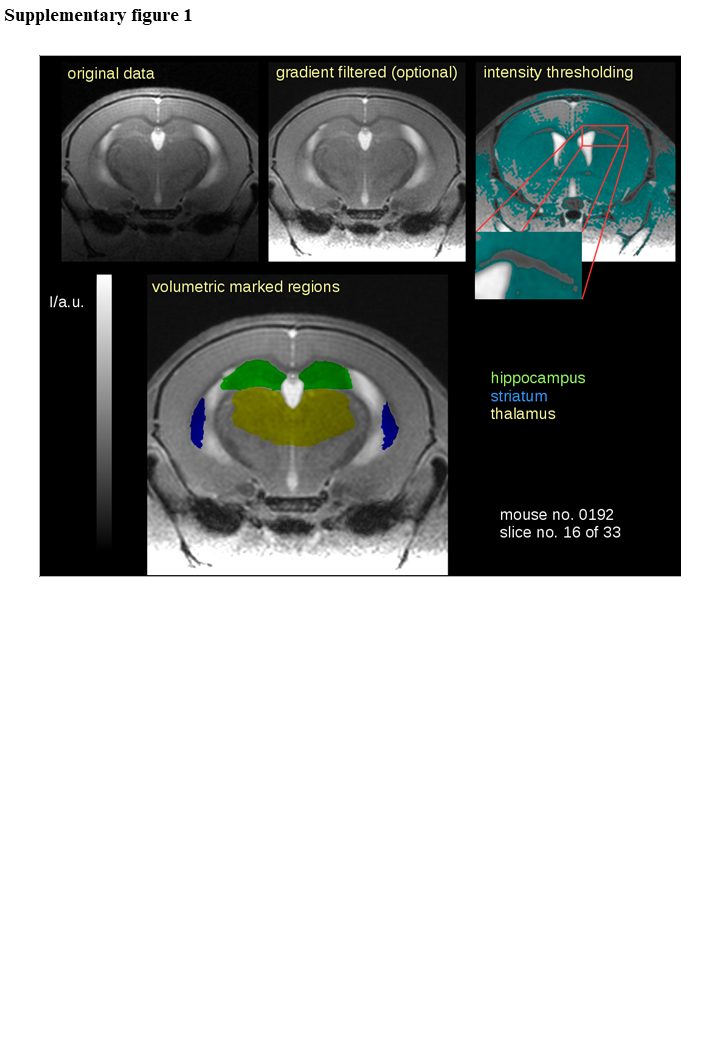

Supplement: FIGURE S1 — Volumetric analysis cascade. Original data were optionally gradient filtered and an intensity thresholding supported the user-defined selection of the respective brain region. Volumetric marked regions were then analyzed for the respective slices. [file Image_1.tif]

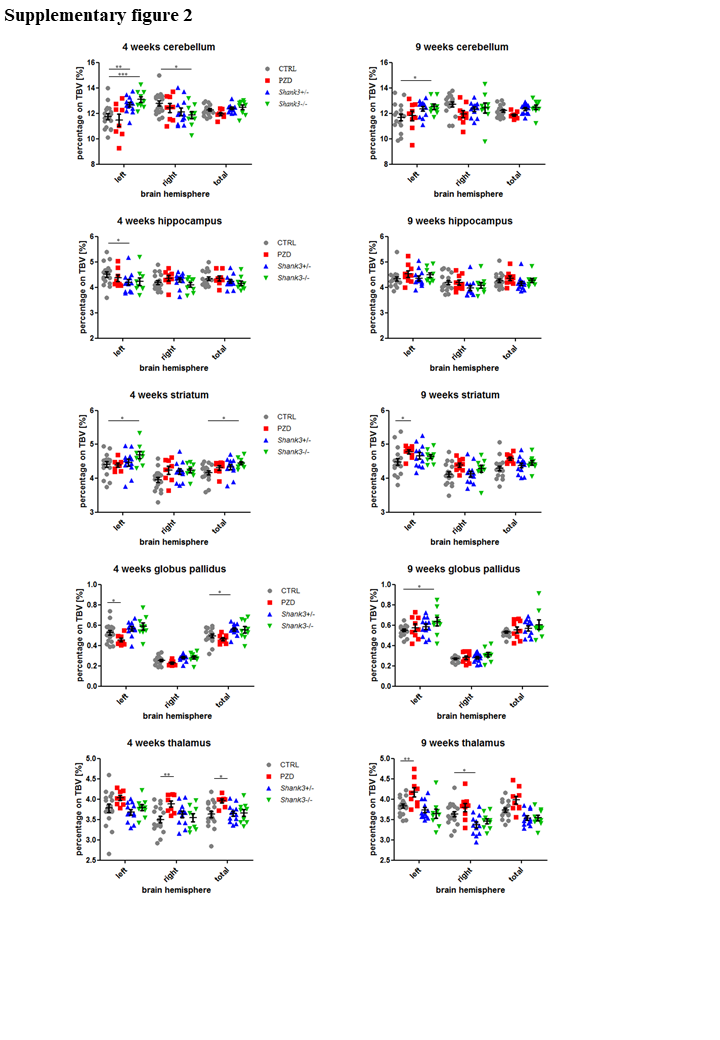

Supplement: FIGURE S2 — Volumes relative to TBV. Each measured brain region (not the cortical thickness) was calculated as the percentage of the total brain volume of the same animal. CTRL, controls; L, left; PZD, prenatal zinc-deficient mice; R, right; Shank3, SH3 and multiple ankyrin repeat domains 3; TBV, total brain volume. The mean values in the diagrams are presented with standard errors of the mean. Significance levels are as follows according to the p-value threshold: < 0.05 = *, < 0.01 = **, < 0.001 = ***. [file Image_2.tif]

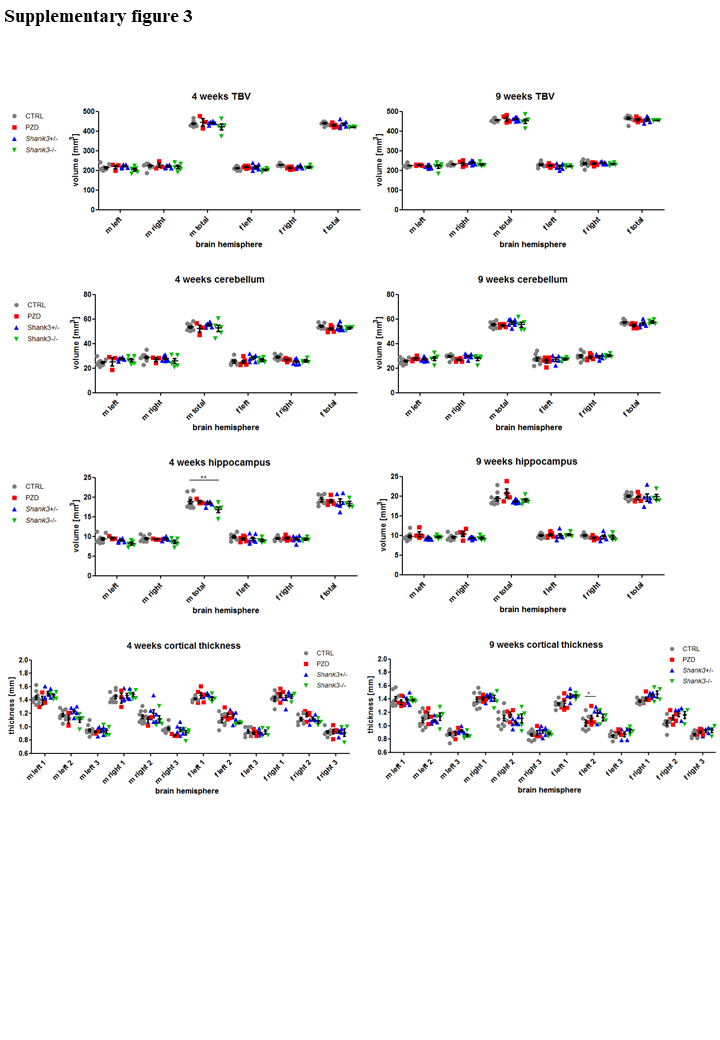

Supplement: FIGURE S3 — Sex differences. Animals were split according to sex. CTRL, controls; f, female; L, left; m, male; PZD, prenatal zinc-deficient mice; R, right; Shank3, SH3 and multiple ankyrin repeat domains 3; TBV, total brain volume. The mean values in the diagrams are presented with standard errors of the mean. Significance levels are as follows according to the p-value threshold: < 0.05 = *, < 0.01 = **, < 0.001 = ***. [file Image_3.tif]

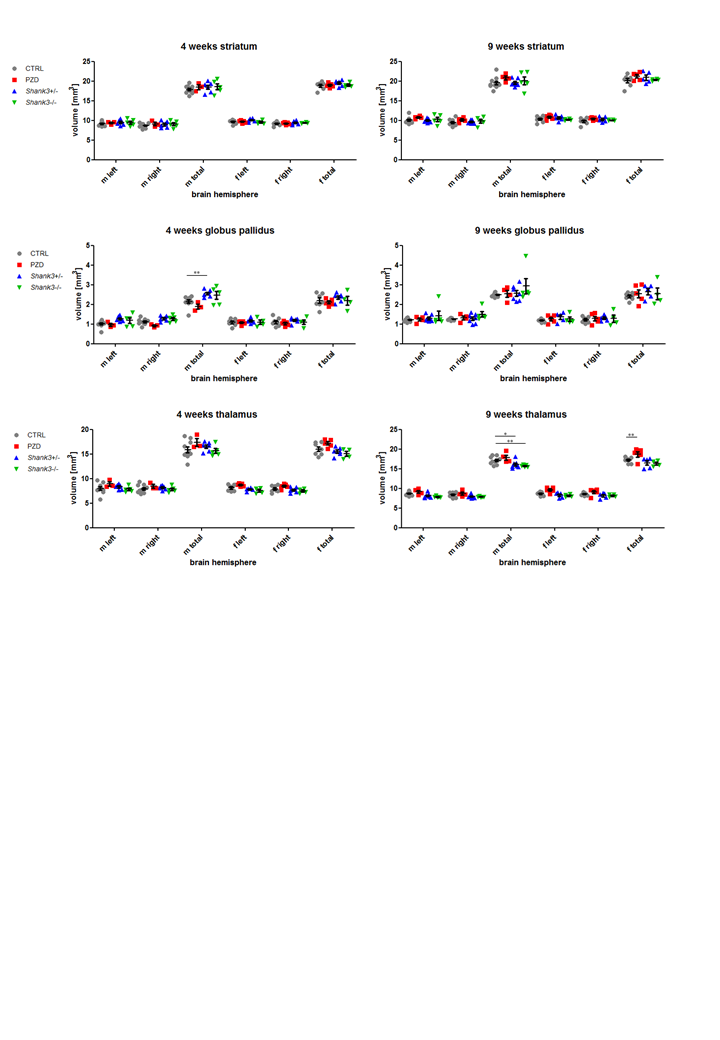

Supplement: Supplementary file 4 [file Image_4.tif]
